# Prediction of ventilator weaning failure in postoperative cardiac surgery patients using vasoactive-ventilation-renal score and nomogram analysis

**DOI:** 10.3389/fcvm.2024.1364211

**Published:** 2024-03-14

**Authors:** Zhongqi Zhang, Wanchun Tang, Yankang Ren, Yifan Zhao, Jinjin You, Han Wang, Sheng Zhao, Xiangrong Zuo

**Affiliations:** ^1^ Department of Critical Care Medicine, The First Affiliated Hospital of Nanjing Medical University, Nanjing, China; ^2^ Department of Cardiovascular Surgery, The First Affiliated Hospital of Nanjing Medical University, Nanjing, China

**Keywords:** cardiac surgery, mechanical ventilation, predictive modelling, vasoactive-inotropic score, vasoactive-ventilation-renal score, weaning failure

## Abstract

**Objective:**

This study evaluated the predictive value of the vasoactive-ventilation-renal (VVR) score in identifying the risk of weaning failure after cardiac surgery and developing a nomogram model to help physicians improve the success rate of weaning from mechanical ventilation in adult patients undergoing postoperative cardiac surgery.

**Methods:**

Clinical data were retrospectively collected from adult patients who underwent extracorporeal circulation cardiac surgery at the First Affiliated Hospital of Nanjing Medical University between August 2022 and April 2023 and who were subsequently transferred to the Intensive Care Unit (ICU) and treated with vasoactive drugs. Patients were divided into successful and unsuccessful weaning groups based on first-attempt weaning success. Variable selection was regularized using univariate logistic regression and Least absolute shrinkage and selection operator (LASSO) regularization. Multivariate logistic regression was performed to identify predictors and a nomogram was created to predict the risk of weaning failure.

**Results:**

A total of 519 patients were included in the study. After selecting multiple stepwise variables, the VVR score before weaning, the modified Sequential Organ Failure Assessment (mSOFA) score on weaning day, and mechanical ventilation duration before weaning were determined as predictive indicators of weaning failure in adult patients after cardiac surgery. The optimal cut-off values for these indicators were 18.46 points, 4.33 points, and 20.50 h, respectively. The predictive model constructed using these three factors demonstrated good predictive performance.

**Conclusions:**

The VVR score before weaning accurately predicts the probability of weaning failure in adult patients after cardiac surgery. The weaning risk-predictive nomogram model, established based on the VVR score, mSOFA score, and mechanical ventilation duration before weaning, demonstrated robust predictive ability.

## 1 Introduction

Mechanical ventilation is one of the most important support measures for patients undergoing post-cardiac surgery. The timing of weaning is crucial, as early weaning or weaning failure can lead to weaning-related heart failure, pulmonary edema, increased rates of reintubation, and mortality (1), Conversely, delay in weaning may lead to pulmonary infections, prolonged mechanical ventilation, prolonged stays in the intensive care unit (ICU), and poor prognoses, among other complications (2). Thus, choosing an appropriate weaning time can help reduce the complications associated with mechanical ventilation, shorten hospital stay, and alleviate the financial burden on patients.

A multicenter retrospective study conducted in 2022 found that the risk of failure of weaning was associated with the dose of vasopressors administered during weaning. When high doses (>0.1 µg/kg/min of norepinephrine equivalents) are administered, the risk of re-intubation significantly exceeds that in patients who use low-dose vasopressor medication (3). Post-cardiac surgery patients have unique circumstances, they often require support from various vasoactive drugs or inotropic substances for a certain time after surgery. If they completely meet the standards of the spontaneous breathing trial (SBT) by discontinuing vasoactive drugs (4), the duration of mechanical ventilation would be significantly prolonged. Therefore, after cardiac surgery, patients usually undergo a SBT and removal of the tracheal tube while receiving vasoactive drugs. According to the literature, the weaning failure rate in patients undergoing post-cardiac surgery varies significantly, ranging from 2.6%–22.7%, one of the most common risk factors for weaning failure is the need for vasoactive drugs (5). Therefore, we believe that the dose of vasoactive drugs used during weaning is crucial for weaning outcomes in patients after cardiac surgery.

In clinical practice, vasoactive drugs commonly used include epinephrine, norepinephrine, dopamine, and dobutamine. The choice of medication typically depends on the patient's condition and the experience of the physician, with slight variations in usage across different treatment centers. As a result, directly comparing the doses of different drugs to gauge the level of hemodynamic support a patient requires can pose a challenge. Therefore, to address this issue, researchers have calibrated the dosages of various vasoactive drugs using a formula known as the Vasoactive Inotropic Score (VIS), effectively quantifying the level of hemodynamic support. It was first proposed by Gaies et al. (6) and has predominantly been used in clinical research for the prognostic analysis of post-cardiac surgery outcomes in infants and adults (7–10), a higher VIS typically signifies more instability in hemodynamics, and hence, a poorer prognosis. However, its predictive capabilities may be suboptimal for postoperative patients with hemodynamically stable conditions or for those with other organ system diseases that can affect prognosis. To address this limitation, Miletic et al. (11) developed the vasoactive ventilation renal (VVR) score, which adds postoperative lung and kidney function parameters to the VIS. The VVR score has been proven to be a stronger predictor of negative post-cardiac surgery outcomes than the VIS, with most adverse outcomes involving extended mechanical ventilation (12–14), however, it is still unclear whether the VVR score can predict the success of weaning and the optimal cutoff values. To provide insights into this issue, a retrospective analysis was conducted to explore the predictive performance of the VVR score in assessing the weaning risk among adult patients subsequent to post-cardiac surgery.

## 2 Methods

### 2.1 Patient population

This was a single-center retrospective observational study. Adult patients who underwent cardiac extracorporeal circulation surgery at the First Affiliated Hospital of Nanjing Medical University between August 2022 and April 2023 were enrolled. This study was approved by the Ethics Committee of the First Affiliated Hospital of Nanjing Medical University (approval number: 2023-SR-380) and the requirement for informed consent was waived.

The inclusion criteria of the study subjects were: (1) age ≥18 years old; (2) patients undergoing cardiovascular surgery, including coronary artery bypass grafting, valve replacement or repair, ascending aorta or aortic arch replacement surgery, congenital heart disease correction; (3) post-surgery transfer to the ICU and continuation of mechanical ventilation, continued use of vasoactive drugs before weaning; and (4) survival of more than 24 h post-surgery.

The exclusion criteria were as follows: (1) patients with a history of chronic pulmonary disease; (2) severely obesity: body mass index (BMI) ≥ 35 kg/m^2^; (3) death before the first attempted weaning after surgery; (4) severe low cardiac output syndrome (A cardiac index of <2.0 L/min/m^2^, along with its clinical signs and symptoms, include hypotension, tachycardia, metabolic acidosis, mixed venous blood oxygen saturation <65%, pallor, cool extremities, pulmonary congestion, and hypoxemia.); (5) Patients who cannot undergo a SBT and directly proceed to tracheostomy; (6) patients with central nervous system complications; and (7) patients with incomplete clinical data.

### 2.2 Data collection and variable definition

The clinical data of the patients was collected and organized, including general information such as sex, BMI, age, smoking and drinking history, and medical history. Preoperative baseline characteristics within 3 days before surgery included cardiac biomarkers, N-terminal pro-B-type natriuretic peptide (NT-proBNP), left ventricular ejection fraction (EF), blood routine, liver and kidney function, D-2 dimer. Operation information included the surgical approach, duration of surgery (recording the time from tracheal intubation to the end of surgery), aortic cross-clamp time, cardiopulmonary bypass time, blood loss, fluid input and output, the intraoperative VIS. Clinical data before weaning included the modified Sequential Organ Failure Assessment (mSOFA) on the day of weaning, liver and kidney function, blood routine, D-2 dimer, cardiac biomarkers, NT-proBNP, the highest level of lactate before weaning, vital signs parameters before weaning, the duration of mechanical ventilation before the first weaning; the VIS and VVR score at the time of ICU admission, the highest level before weaning, and at the time of weaning. All clinical information was obtained from the hospital's electronic medical record system and ICU Nursing Record Sheet.

mSOFA (3): the sum of respiratory, coagulation, liver, neurological, and kidney sections. (Considering the relevance of the cardiovascular section to the treatment with vasoactive drugs, this part was omitted).

Prognostic Nutritional Index (PNI) (10) = 5 × peripheral blood lymphocyte count (×10^9^/L) + serum albumin concentration (g/L). This index is used to assess the nutritional status of patients before surgery and to predict postoperative complications. A low PNI score indicated malnutrition or a high risk of complications.

The VIS (6): VIS = Dopamine [µg/(kg·min)] + dobutamine [µg/(kg·min)] + 10 × milrinone [µg/(kg·min)] + 100 × epinephrine [µg/(kg·min)] + 100 × norepinephrine [µg/(kg·min)] + 10,000 × vasopressin [U/(kg·min)].

The VVR score (13): the VVR = VIS + Ventilation Index Score + (ΔCr × 10).

To calculate the Ventilation Index Score patient parameters were collected, such as the arterial blood carbon dioxide partial pressure (PaCO_2_), respiratory rate (RR), peak airway pressure (PIP), and positive end-expiratory pressure (PEEP). Ventilation Index Score = (Ventilator RR) × (PIP-PEEP) × PaCO_2_/1,000.

To calculate the ΔCr, the baseline (pre-operative) serum creatinine value was subtracted from the postoperative serum creatinine value and denoted as ΔCr. The postoperative serum creatinine level refers to the level of creatinine in the blood measured on the day that the VVR score is calculated. Creatinine levels were expressed in mg/dl. Creatinine was measured in µmol/L and was converted to mg/dl using the following calculation formula: 1 mg/dl = 88.4 µmol/L, for patients whose postoperative serum creatinine value was less than or equal to the baseline, ΔCr = 0.

### 2.3 Weaning procedure

After cardiac surgery, all adult patients were routinely transferred to the ICU for sedation, analgesia, mechanical ventilation, and continuous electrocardiographic and hemodynamic monitoring. Postoperatively, patients received intravenous vasoactive drugs, such as epinephrine, dopamine, and milrinone. Mechanical ventilation was performed using the pressure control synchronized intermittent mandatory ventilation (P-SIMV) mode, with an I/E ratio of 1:2, support pressure of 10–20 cmH_2_O (maintaining a tidal volume of approximately 8 ml/kg), PEEP of 5 cm H_2_O, a respiratory rate of 12–15 breaths/min, and FiO_2_ of 40%–60% (maintaining SpO_2_ ≥ 95%). For patients who meet the weaning criteria, initiate a SBT. General requirements are as follows: The patient is awake and cooperative, had no active bleeding (chest tube drainage ≤ 100 ml/h), and hemodynamics are stable (ScvO_2_ > 65%, CI > 2.2 L/min/m^2^, MAP > 65 mmHg with low-dose epinephrine ≤0.2 µg/kg/min), no signs of pericardial effusion or significant heart failure on bedside echocardiography, improved respiratory function (PSV 7–10 cmH_2_O, FiO_2_ ≤ 50%, PEEP ≤ 5–8 mmHg, PaO_2_/FiO_2_ ≥ 200), no obvious electrolyte or acid-base balance disorder, when the first SBT was performed. The SBT was performed for 30 min. If the patient showed one or more of the following signs at the end of the first SBT, the SBT was considered to have failed (15): RR > 35 times/min or an increase of ≥50%; HR > 140 beats/min or an increase of ≥20%; SpO_2_ < 90% or PaO_2_ < 60 mmHg; respiratory acidosis (pH < 7.3 or PaCO_2_ > 50 mmHg); signs of respiratory distress, such as asynchronized chest and abdominal movements, anxiety and sweating, and new-onset arrhythmia.

### 2.4 Outcome definition

The primary outcome was success or failure of weaning. Successful weaning was defined as (16): (1) the patient was transferred to the ICU after surgery. When the patients met the criteria for weaning off the ventilator, a SBT of 30 min was conducted and if successful, the endotracheal tube was removed and mechanical ventilation was stopped; (2) the patient did not require re-intubation or resumption of noninvasive ventilation support within 48 h after weaning; (3) the patient was alive 48 h after extubation. Weaning failure was defined as one of the following (16): (1) the patient could not maintain effective ventilation or oxygenation on their own, and needed to be re-intubated for mechanical ventilation within 48 h after extubation; (2) the patient did not pass the SBT; (3) the patient resumed non-invasive ventilation support within 48 h after extubation; or (4) the patient died within 48 h after extubation.

### 2.5 Development of the nomogram

Univariate logistic regression was used to evaluate the predictive variables, with further selection of significant variables using the Least absolute shrinkage and selection operator (LASSO) regularization method (17). Subsequently, factors with nonzero coefficients in the LASSO regression were used by performing bidirectional selection of multivariable logistic regression to develop the prediction model and nomogram.

Considering the differences between various surgeries, the predictive precision and consistency of the model were evaluated separately in the coronary artery bypass grafting and cardiac valve surgery subgroups.

### 2.6 Statistical analysis

Statistical analyses were conducted using SPSS v25.0. The Shapiro–Wilk test was used for normality testing of the data. Quantitative data that followed a normal distribution were presented as mean ± standard deviation (mean ± SD), and the *t*-test was used for comparison between groups. Non-normally distributed quantitative data were presented as median (interquartile range) [M (QL, QU)], and the Mann–Whitney-*U*-test was used for comparison between groups. Count data were represented as rates, with a comparison between groups conducted using the *χ*^2^ test. A nomogram model was established using R 4.3.0 software. Internal validation of the model was performed using the bootstrap resampling method with 1,000 repeats, and the concordance index (C-index) was used to measure the accuracy of the model. The discrimination and calibration of the model was performed using the area under the receiver operating characteristic curve (ROC-AUC) and the calibration curves. A decision curve analysis (DCA) was implemented to evaluate clinical usefulness and net benefit. Statistical significance was established at a *P*-value <0.05. All statistical analyses were performed using R 4.3.0 software (The R Foundation for Statistical Computing, Vienna, Austria) with rms, glmnet, pROC, and dca packages.

## 3 Results

### 3.1 General characteristics

**Figure 1:**
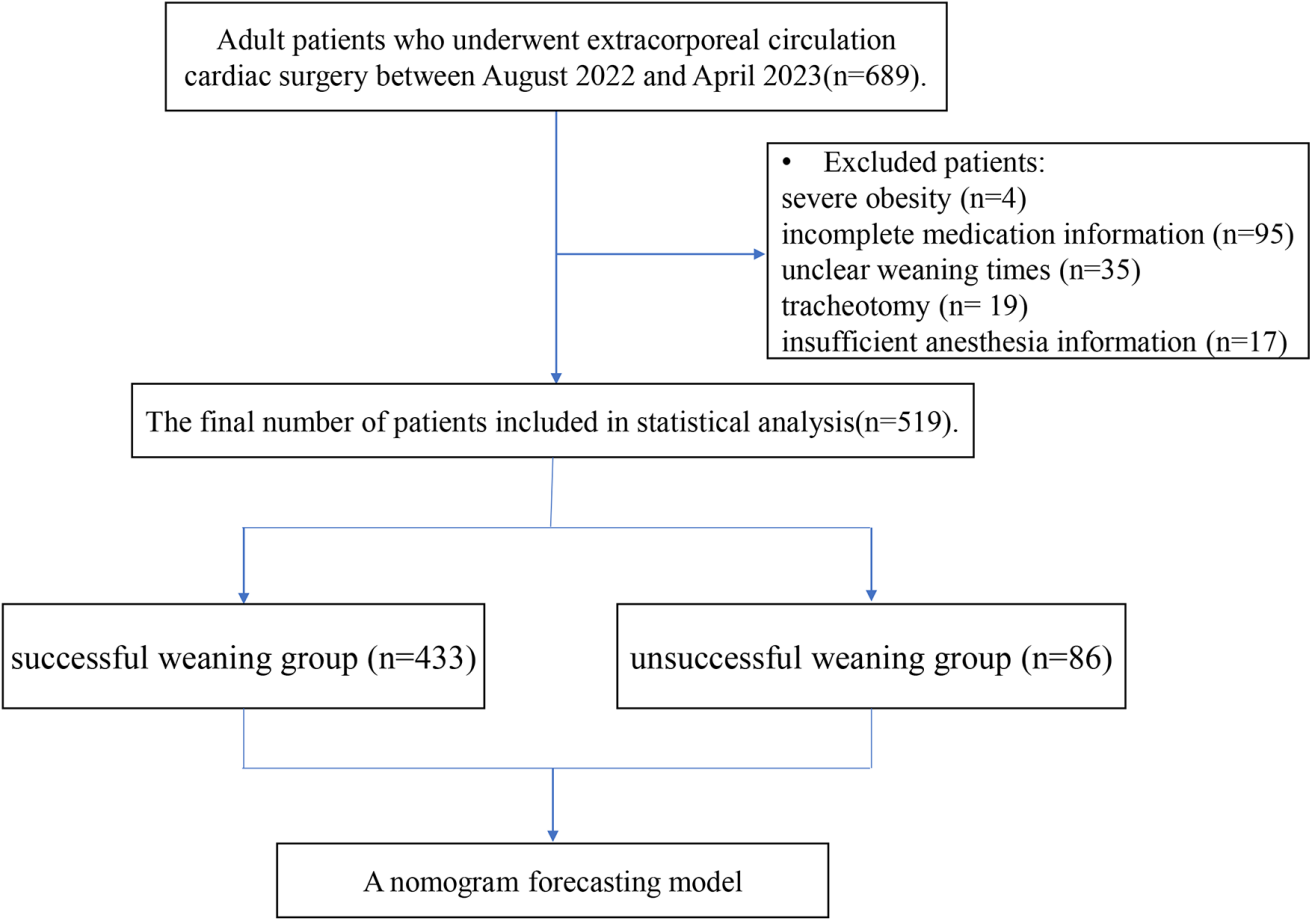
Research process flowchart.

During the research period (August 2022–April 2023), 689 adult patients undergoing cardiac surgery were evaluated for eligibility. Of these, 170 patients were excluded because of severe obesity (4 patients), incomplete medication information (95 patients), unclear weaning times (35 patients), tracheotomy need (19 patients), or insufficient anesthesia information (17 patients). Ultimately, 519 patients were included in this study (Figure 1), with weaning failure events occurring in 86 (16.6%). Of these, 64 (12.3%) failed the SBT, 13 (2.5%) shifted to non-invasive ventilation within 48 h post-weaning, and 9 (1.7%) required re-intubation within 48 h post-surgery. The general baseline characteristics of the patients are shown in Table 1.

**Table 1:** Comparison of clinical data between two groups of patients undergoing mechanical ventilation after cardiac surgery.

Variables	Successful weaning group	Failed weaning group	*P*
Demographics			
Sex (male)	271 (62.60)	53 (61.60)	0.867
Age (year)	60.00 (54.00,68.00)	64.00 (57.00,69.00)	0.052
BMI (kg/m^2^)	24.14 ± 3.00	23.88 ± 3.37	0.473
Smoking history	157 (36.30)	29 (33.70)	0.654
Drinking history	116 (26.80)	15 (17.40)	0.068
Underlying conditions			
Coronary heart disease	178 (41.10)	35 (40.70)	0.944
Atrial fibrillation	89 (20.60)	21 (24.40)	0.423
Valvular heart disease	276 (63.70)	56 (65.10)	0.808
Aortic dissection	25 (5.80)	9 (10.50)	0.108
Ascending aortic dilation	37 (8.05)	6 (7.00)	0.630
Congenital heart disease	35 (8.10)	4 (4.70)	0.270
Hypertension	174 (40.20)	38 (44.20)	0.491
Diabetes	74 (17.10)	8 (9.30)	0.071
Chronic kidney disease	14 (3.20)	2 (2.30)	0.656
Cerebral infarction	79 (18.20)	16 (18.60)	0.937
Chronic liver disease	8 (1.80)	2 (2.30)	0.768
Preoperative information			
WBC count (10^9^/L)	6.11 (4.78,7.35)	6.20 (5.12,7.06)	0.616
Plt count (10^9^/L)	193.38 ± 61.06	196.37 ± 54.62	0.673
PNI	47.96 ± 6.51	47.25 ± 8.74	0.384
Hemoglobin (g/L)	136.00 (122.00,145.00)	133.00 (125.00,140.00)	0.146
D-2 polymer (mg/L)	0.30 (0.17,0.54)	0.35 (0.19,0.72)	0.150
Troponin T (ng/L)	12.56 (9.33,18.66)	12.51 (11.07,23.36)	0.437
Serum NT-proBNP (pg/ml)	1,672.43 ± 4,031.34	2,679.09 ± 5,504.50	0.110
Left ventricular ejection fraction (%)	62.10 (59.00,64.00)	60.40 (52.63,63.60)	0.013
Surgical types			
Coronary artery bypass graft surgery	169 (39.00)	36 (41.90)	0.624
Valve surgery	260 (60.00)	54 (62.80)	0.634
Ascending aorta or aortic arch replacement	60 (13.90)	17 (19.80)	0.159
Coronary artery bypass graft surgery + valve surgery	30 (6.90)	11 (12.80)	0.066
Corrective surgery for congenital heart disease	26 (6.00)	3 (3.50)	0.353
Intraoperative information			
Intraoperative bleeding volume (ml)	600.00 (500.00,800.00)	650.00 (500.00,800.00)	0.251
Surgical duration (h)	6.27 ± 1.54	7.05 ± 1.58	<0.001
Aortic cross-clamp time (h)	1.67 (1.22,2.05)	1.81 (1.40,2.33)	0.066
Cardiopulmonary bypass time (h)	2.20 (1.72,2.78)	2.45 (1.85,2.94)	0.165
Total intraoperative fluid input (ml)	4,075.76 ± 1,619.67	4,621.21 ± 1,507.89	0.004
Total intraoperative fluid output (ml)	3,083.55 ± 1,543.90	3,620.56 ± 1,660.22	0.004
VIS at the beginning of the surgery (score)	3.00 (3.00,5.00)	3.00 (3.00,5.00)	0.637
VIS at the end of the surgery (score)	7.00 (3.00,8.00)	7.00 (4.00,8.00)	0.286
Highest VIS during the surgery (score)	7.00 (3.00,8.00)	7.00 (5.00,9.00)	0.081
Postoperative information			
Postoperative use of recombinant human brain natriuretic peptide	289 (66.70)	62 (72.10)	0.333
Postoperative use of nitroglycerin	205 (47.30)	31 (36.00)	0.055
Mechanical ventilation duration before weaning (h)	15.00 (7.00,20.00)	25.00 (19.00,65.00)	<0.001
Serum NT-proBNP on weaning day (pg/ml)	1,394.90 (772.40,2,079.80)	2,294.00 (1,094.70,5,231.00)	<0.001
Highest lactate level before weaning (mmol/L)	3.50 (2.00,5.20)	4.55 (2.60,6.20)	0.008
mSOFA on weaning day (score)	3.37 ± 1.48	4.65 ± 1.58	<0.001
WBC count on weaning day (10^9^/L)	11.56 (9.41,14.51)	11.55 (9.32,14.94)	0.976
Hemoglobin on weaning day (g/L)	103.00 (93.00,117.00)	99.50 (89.00,110.00)	0.009
D-dimer on weaning day (mg/L)	1.78 (1.00,3.14)	2.18 (1.12,3.99)	0.015
Troponin T on weaning day (ng/L)	420.50 (190.00,862.40)	552.40 (213.10,1,314.00)	0.067
Procalcitonin on weaning day (ng/ml)	0.68 (0.33,1.45)	1.33 (0.33,3.04)	0.001
Fastest heart rate before weaning (beats/min)	88.00 (80.00,96.00)	88.00 (83.00,99.00)	0.283
Highest mean arterial pressure before weaning (mmHg)	87.00 (79.00,94.00)	87.00 (81.00,94.00)	0.577
Highest central venous pressure before weaning (cmH_2_O)	10.00 (7.00,12.00)	10.00 (8.00,12.00)	0.224
VIS on admission to ICU (score)	7.00 (3.60,11.20)	9.10 (7.00,13.90)	<0.001
VIS before weaning (score)	6.63 (4.70,10.00)	11.80 (8.50,13.80)	<0.001
Highest VIS before weaning (score)	11.10 (7.00,17.10)	17.85 (13.40,25.20)	<0.001
VVR on admission to ICU (score)	15.33 (11.81,19.89)	17.66 (14.92,21.90)	<0.001
VVR before weaning (score)	14.53 (11.51,17.62)	20.41 (16.81,23.99)	<0.001
Highest VVR before weaning (score)	19.70 (15.65,25.62)	26.93 (21.93,36.50)	<0.001

BMI, body mass index; WBC, white blood cell; PNI, prognostic nutritional index; NT-proBNP, N-terminal pro-B-type natriuretic peptide; mSOFA, modified sequential organ failure assessment; VIS, vasoactive-inotropic score; VVR, vasoactive-ventilation-renal score.

Within the baseline table, there were differences in the VIS and VVR score over various time intervals. The area under the ROC curve is presented in Table 2. The ROC curve area under the VVR score at different stages was higher than that of the VIS, indicating that the predictive power of the VVR score for weaning failure in post-cardiac surgery patients was superior to that of the VIS (Supplementary Figure S1). Among these, the VVR score immediately before weaning had the highest predictive accuracy for weaning failure, with an area under the ROC curve of 0.793.

**Table 2:** Predictive value of VIS and VVR for weaning failure in patients after cardiac surgery.

Variables	AUC	*P*	95% CI	
			Lower limit	Upper limit
VIS on admission to ICU (score)	0.633	<0.001	0.576	0.690
VIS before weaning (score)	0.751	<0.001	0.695	0.807
Highest VIS before weaning (score)	0.718	<0.001	0.664	0.772
VVR on admission to ICU (score)	0.636	<0.001	0.579	0.693
VVR before weaning (score)	0.793	<0.001	0.740	0.845
Highest VVR before weaning (score)	0.750	<0.001	0.700	0.800

AUC, area under the receiver operating characteristic curve; CI, confidence interval; VIS, vasoactive-inotropic score; VVR, vasoactive-ventilation-renal score.

### 3.2 Screening for predictive factors

**Figure 2:**
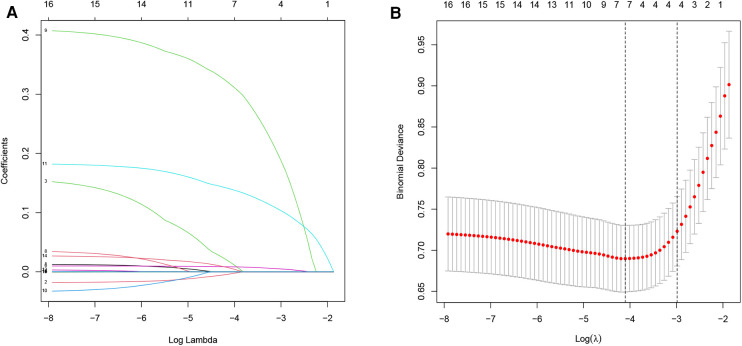
Variable selection via LASSO regression. (**A**) LASSO coefficient profiles from univariate analysis of variable significance, with each coefficient plot generated against a sequence of log (λ) values. (**B**) Selection of seven non-zero coefficient variables at the optimal lambda. The relationship between partial likelihood deviance (binomial deviance) curves and log (λ) is plotted to validate the optimal parameter (λ) in the LASSO model, with vertical dashed lines set at the minimum criteria and one standard error above the minimum criterion.

According to the results of ROC curve analysis, the VVR score outperformed the VIS in terms of predictive capability; hence, we incorporated the VVR score as a predictive factor for further selection. Through univariate analysis (Supplementary Table S1), we identified 16 significant predictors of weaning failure (*P *< 0.05) among the 55 incorporated variables. Next, we used the LASSO algorithm based on each predictor for further variable selection (Figure 2). Of these 16 variables, the LASSO algorithm chose seven potential predictors with nonzero coefficients, which included preoperative D-2 dimer, left ventricular ejection fraction, mechanical ventilation duration before weaning, the mSOFA on weaning day, serum NT-proBNP on weaning day, surgical duration, and the VVR score before weaning. Subsequently, we conducted a multivariate logistic regression analysis to identify predictors of weaning failure. According to the results in Table 3, the VVR score before weaning, duration of mechanical ventilation before weaning, and the mSOFA score on the day of weaning were predictors of weaning failure.

**Table 3:** Multivariable logistic regression analysis after LASSO.

Predictive factors	OR	95% CI	*P*
VVR before weaning (score)	1.182	1.124–1.244	<0.001
mSOFA on weaning day (score)	1.480	1.224–1.790	<0.001
Mechanical ventilation duration before weaning (h)	1.010	1.004–1.016	<0.001
Serum NT-proBNP on weaning day (pg/ml)	1.000	0.999–1.001	0.143
Left ventricular ejection fraction (%)	0.983	0.954–1.013	0.272
D-2 polymer (mg/L)	1.026	0.973–1.082	0.342
Surgical duration (h)	1.080	0.906–1.286	0.392

OR, odds ratio; CI, confidence interval; VVR, vasoactive-ventilation-renal score; NT-proBNP, N-terminal pro-B-type natriuretic peptide; mSOFA, modified sequential organ failure assessment.

### 3.3 Determination of the optimal cut-off value

The results of the ROC curve analysis are shown in Table 4. The AUCs for predicting weaning failure for the preweaning VVR score, mechanical ventilation duration before weaning, and the mSOFA score on weaning day were 0.793 (95% CI: 0.740–0.845), 0.738 (95% CI: 0.662–0.782), and 0.722 (95% CI: 0.662–0.782), respectively (Supplementary Figure S2). Calculated using the maximum Youden index, the optimal cut-off values for the VVR score, mechanical ventilation duration, and the mSOFA were 18.46 (sensitivity: 0.698, specificity: 0.801), 20.50 (sensitivity: 0.709, specificity: 0.758), and 4.33 (sensitivity: 0.547, specificity: 0.774), respectively. Based on a comprehensive evaluation, the VVR score before weaning had the highest predictive capability of weaning failure in patients after cardiac surgery.

**Table 4:** ROC analysis of VVR before weaning, mechanical ventilation duration before weaning, and mSOFA on weaning day for predicting weaning failure in post-cardiac surgery patients.

Variables	AUC	*P*	Optimal cut-off value	95% CI		Sensitivity	Specificity
				Lower limit	Upper limit		
VVR before weaning (score)	0.793	<0.001	18.46	0.740	0.845	0.698	0.801
Mechanical ventilation duration before weaning (h)	0.738	<0.001	20.50	0.662	0.782	0.709	0.758
mSOFA on weaning day (score)	0.722	<0.001	4.33	0.662	0.782	0.547	0.774

AUC, area under the receiver operating characteristic curve; CI, confidence interval; VVR, vasoactive-ventilation-renal score; mSOFA, modified sequential organ failure assessment.

### 3.4 Development and validation of the nomogram

**Figure 3:**
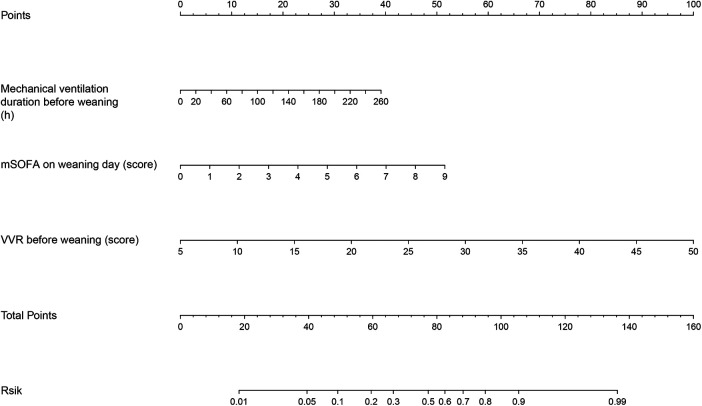
Nomogram for predicting the failure of weaning from mechanical ventilation in post-cardiac surgery patients. mSOFA, modified sequential organ failure assessment; VVR, vasoactive-ventilation-renal score.

We collected data from 519 adult patients who met the sample size requirements for constructing a nomogram forecasting model based on the three factors mentioned above. Considering that data classification loses its statistical power, we did not transform the data into binary variables. Subsequently, we integrated and constructed a nomogram (Figure 3) to predict the risk of weaning failure based on these three factors. The scores for these factors were assigned by drawing a vertical line from the corresponding value to the “Points” line; the sum of the points for the three items was plotted on the “Total Points” line. Finally, a vertical line was drawn downward to determine the risk of weaning failure after cardiac surgery.

**Figure 4:**
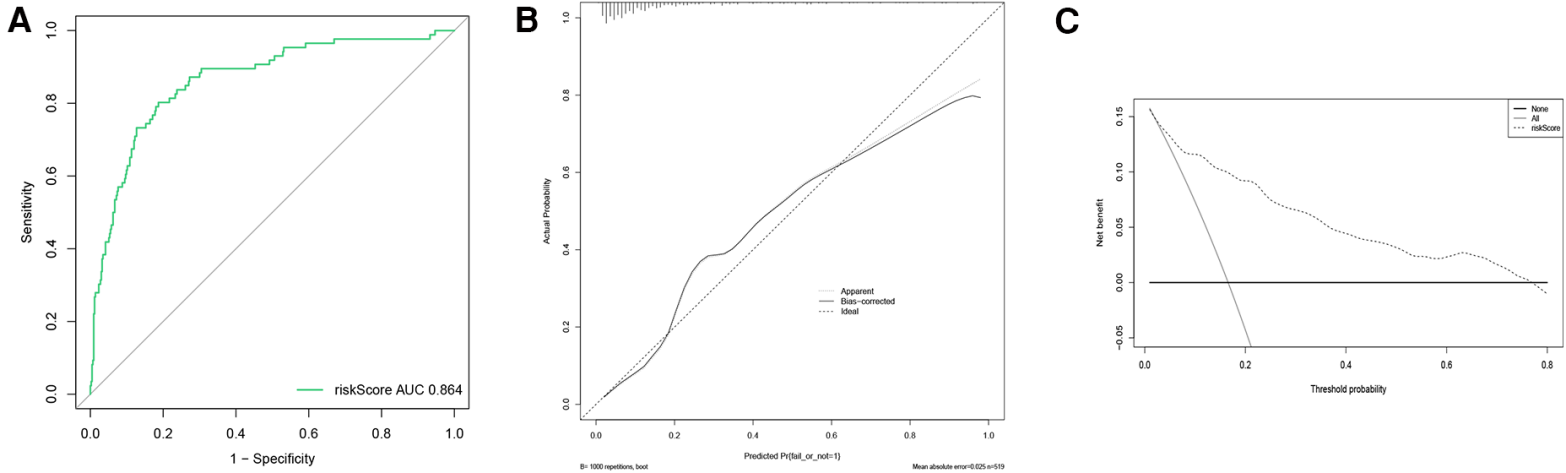
ROC curve, calibration curve, and DCA curve for the nomogram predicting weaning failure. (**A**) ROC curve; (**B**) Calibration curve; (**C**) DCA curve. AUC represents the area under the ROC curve.

The discrimination of the nomogram was assessed using the C-index, which was 0.864 [95% CI (0.818–0.908)], demonstrating good accuracy. The ROC-AUC = C-index = 0.864 [95% CI (0.818–0.908)]. After internal validation of the nomogram model using the bootstrap resampling method 1,000 times, a high-quality prediction model calibration curve was obtained, indicating good consistency between the prediction model and the actual observed results. Moreover, the area under the DCA showed the clinical utility of this prediction model, indicating that our nomogram had the potential for clinical decision-making (Figure 4).

### 3.5 Subgroup analysis

In the population included in this study, 147 patients underwent only coronary artery bypass grafting and 218 patients underwent only cardiac valve surgery. The probabilities of weaning failure were 13.6% (20) and 15.6% (34), respectively. The *P*-value is 0.599, indicating no statistically significant difference. Among the three indicators used to construct the nomogram, the ROC-AUC of the VVR score before weaning in patients after coronary artery bypass surgery was 0.783 [95% CI (0.663–0.903)], the mSOFA was 0.774 [95% CI (0.665–0.884)], and the mechanical ventilation duration was 0.677 [95% CI (0.551–0.804)]. The ROC-AUC of the VVR score before weaning in patients after cardiac valve surgery was 0.853 [95% CI (0.793–0.914)], that of the mSOFA was 0.754 [95% CI (0.669–0.839)], and that of mechanical ventilation duration was 0.775 [95% CI: 0.695–0.854)]. The VVR score showed better predictive performance in patients after cardiac valve surgery. Furthermore, our nomogram demonstrated good predictive power for weaning failure in both the coronary artery bypass grafting group and the cardiac valve surgery group. The ROC-AUCs were 0.878 [95% CI (0.776– 0.979)] and 0.897 [95% CI (0.845–0.949)], respectively. The calibration curve also showed good consistency between the predicted and actual probabilities of weaning failure, and the DCA curve in the two subgroups confirmed the clinical value of the prediction model (Supplementary Figure S3).

## 4 Discussion

In this retrospective study involving 519 patients, we found that 16.6% of postoperative cardiac patients experienced weaning failure, which was similar to previous findings (19.4%) (18). We established a clinical prediction model for weaning failure in patients undergoing cardiac surgery using preweaning variables. Through multistep variable selection of logistic regression and LASSO regularization, we found that VVR score before weaning, mechanical ventilation duration before weaning, and mSOFA score on the weaning day were predictors of post-cardiac surgery weaning. Among these, the VVR score before weaning had the best predictive performance, with an AUC-ROC of 0.793. The optimal cut-off value was 18.46, with a sensitivity of 0.698 and a specificity of 0.801. We developed and validated a nomogram to predict weaning failure based on these three predictors, and further evaluated the value of the nomogram in subgroups after coronary artery bypass grafting and cardiac valve surgery.

The VVR score, which includes the VIS, Ventilation Index Score, and preoperative and postoperative changes in creatinine levels, is considered a reliable predictor of prognosis in pediatric patients after congenital heart surgery. The predictive performance of the VVR score is superior to that of the VIS (19), although the VIS has been shown to predict the prognosis of patients undergoing post-cardiac surgery, primarily reflecting the patient's postoperative cardiovascular condition and does not consider the impact of other functions of the organ system on the patient. Therefore, the VVR score, which includes indicators of cardiovascular, respiratory, and renal function, can better reflect the burden on various systems in patients after cardiac surgery. In a study by Cashen et al., the researchers found that the highest VVR score at 12 h after surgery (AUCROC = 0.82) was a better predictor of the duration of prolonged mechanical ventilation in neonatal patients after cardiac surgery than individual ventilation index scores (AUCROC = 0.78) and VIS (AUCROC = 0.70) (14), which is consistent with the results of our study.

The underlying cause of weaning failure may be that patients who undergo cardiac surgery often have ischemic heart disease, valvular heart disease, and systolic or diastolic heart dysfunction prior to surgery. During routine weaning, the transition from positive pressure ventilation to spontaneous breathing results in an increase in intrathoracic negative pressure and venous return, leading to an increase in left ventricular pre-load and myocardial oxygen consumption (20). Consequently, heart dysfunction often becomes more apparent during weaning, and a lack of cardiac output can lead to weaning failure. The VVR score before weaning, through its weighted calculation of vasoactive drugs, can represent the cardiac status at the time of weaning preparation to some extent. Furthermore, weaning failure in some patients after cardiopulmonary surgery may be due not only to cardiac insufficiency, but also to extracardiac causes such as respiratory insufficiency, renal dysfunction, neuromuscular ability, neuropsychological factors, and metabolic and endocrine disorders (21). The preweaning VVR score includes indicators related to lung and kidney function prior to weaning. Compared with the single-factor VIS, the VVR score considers a broader range of factors; therefore, its clinical predictive performance is higher.

Successful weaning from mechanical ventilation is influenced by several factors. Through multivariate logistic regression analysis, we found that the duration of mechanical ventilation before the first weaning attempt and the mSOFA score on the day of weaning were independent risk factors for weaning failure in patients with post-cardiac surgery. The SOFA is a widely used assessment tool in the ICU for evaluating the degree of organ dysfunction and prognostication, encompassing the respiration, blood coagulation, liver, circulation, central nervous system, and renal function. A high SOFA score indicated severe organ dysfunction. Previous research has revealed a correlation between the SOFA score and the time required for patients to be released from mechanical ventilation, with dynamic changes in the SOFA score serving as successful predictors of weaning (22). In another study comparing the effects of different weaning methods on weaning success rates (23), the authors identified the baseline SOFA score, the duration of mechanical ventilation before weaning, and the weaning method as predictive factors for weaning success through a multifactorial regression analysis, which is consistent with our current findings. Furthermore, the longer the duration of mechanical ventilation before the first attempt at weaning, the higher the dependency on mechanical ventilation, increases the probability of ventilator-associated pneumonia and making weaning more difficult (24, 25). Based on these three independent risk factors, our study established a nomogram model that can assist clinicians in making early predictions of weaning failure based on individual risk factors, thereby providing guidance for optimal clinical decision-making.

This study had several limitations. First, this was a retrospective observational study. A selection bias may have been present, which may have affected the results. Second, this was a single-center study that assessed only patients undergoing cardiac surgery; therefore, our results may not be applicable to other surgical populations. Third, the VVR score has its own limitations as it does not include all vasoactive drugs, such as nitroglycerin and natriuretic peptide. Fourth, the surgical skills of the physicians, the severity of patient conditions in different medical institutions, the use of vasoactive drugs, and the settings of ventilator-related parameters were affected by the preferences of the clinical physicians, limiting their universality to some extent. Finally, we did not investigate the long-term outcomes of patients due to the limited sample size.

## 5 Conclusions

In postoperative cardiac surgery patients, the VVR score provides a robust predictive measure for weaning failure. A nomogram model incorporating VVR, mSOFA scores, and mechanical ventilation duration offers reliable predictions for weaning outcomes and may assist in clinical decision-making. However, the current findings, derived from a limited single-center study, warrant further validation with a larger, multicenter cohort.

## Data Availability

The raw data supporting the conclusions of this article will be made available by the authors, without undue reservation.
