# Supplementary material for: Prediction of ventilator weaning failure in postoperative cardiac surgery patients using vasoactive-ventilation-renal score and nomogram analysis
Source: Front Cardiovasc Med. 2024 Mar 14;11:1364211. doi: 10.3389/fcvm.2024.1364211 (PMC10977076; doi:10.3389/fcvm.2024.1364211)
Supplement: Supplementary file 1 [file Datasheet1.zip › Data Sheet 1_v1/Supplementary Material.docx]

**Figure S1:** ROC curves for VIS and VVR predicting weaning failure at different stages.

VIS, vasoactive-inotropic score; VVR, vasoactive-ventilation-renal score.

**Figure S2:** ROC curves for VVR before weaning, Mechanical ventilation duration before weaning, and mSOFA on weaning day for predicting weaning failure in post-cardiac surgery patients.

VVR, vasoactive ventilation renal score; mSOFA, modified Sequential Organ Failure Assessment.

**Figure S3:** ROC curves, calibration curves, and DCA curves for the nomogram in predicting weaning failure among patients with coronary artery disease and those undergoing valve surgery. (**A and B**): ROC curves for the cohorts of coronary artery disease and valve surgery patients; (**C and D**): calibration curves for the cohorts of coronary artery disease and valve surgery patients; (**E and F**): DCA curves for the cohorts of coronary artery disease and valve surgery patients. AUC represents the area under the ROC curve.

**Table S1:** Univariate logistic regression for screening predictors of post-cardiac surgery weaning failure.

BMI, Body mass index; WBC, White blood cell; PNI, Prognostic Nutritional Index; NT-proBNP, N-terminal pro-B-type natriuretic peptide; mSOFA, modified Sequential Organ Failure Assessment; VIS, vasoactive-inotropic score; VVR, vasoactive-ventilation-renal score.
