# Supplementary material for: Prediction of ventilator weaning failure in postoperative cardiac surgery patients using vasoactive-ventilation-renal score and nomogram analysis
Source: Front Cardiovasc Med. 2024 Mar 14;11:1364211. doi: 10.3389/fcvm.2024.1364211 (PMC10977076; doi:10.3389/fcvm.2024.1364211)
Supplement: Supplementary file 1 [file Datasheet1.zip › Data Sheet 1_v1/Table S1.docx]

**Table S1:** Univariate logistic regression for screening predictors of post-cardiac surgery weaning failure.

| Variables | OR | 95%CI | | | P |
| --- | --- | --- | --- | --- | --- |
|  |  | Lower Limit | | Upper Limit |  |
| Demographics | | | | | |
| Sex (male) | 0.9601 | 0.5963 | 1.5457 | | 0.8668 |
| Age (year) | 1.0236 | 1.0009 | 1.0469 | | 0.0419 |
| BMI(kg/m^2^) | 0.9727 | 0.9020 | 1.0490 | | 0.4726 |
| Smoking history | 0.8944 | 0.5490 | 1.4572 | | 0.6540 |
| Drinking history | 0.5773 | 0.3181 | 1.0480 | | 0.0709 |
| Underlying conditions | | | | | |
| Coronary heart disease | 0.9831 | 0.6139 | 1.5744 | | 0.9436 |
| Atrial fibrillation | 1.2487 | 0.7245 | 2.1523 | | 0.4238 |
| Valvular heart disease | 1.0618 | 0.6539 | 1.7243 | | 0.8083 |
| Aortic dissection | 1.9075 | 0.8572 | 4.2448 | | 0.1135 |
| Ascending aortic dilation | 0.8027 | 0.3278 | 1.9653 | | 0.6305 |
| Congenital heart disease | 0.5547 | 0.1919 | 1.6033 | | 0.2765 |
| Hypertension | 1.1784 | 0.7388 | 1.8797 | | 0.4908 |
| Diabetes | 0.4976 | 0.2305 | 1.074 | | 0.0754 |
| Chronic kidney disease | 0.7126 | 0.1590 | 3.1937 | | 0.6579 |
| Cerebral infarction | 1.0242 | 0.5647 | 1.8576 | | 0.9372 |
| Chronic liver disease | 1.2649 | 0.2641 | 6.0576 | | 0.7687 |
| Preoperative information | | | | | |
| WBC count (10^9^/L) | 1.0221 | 0.9502 | 1.0994 | | 0.5576 |
| Plt count (10^9^/L) | 1.0008 | 0.9970 | 1.0047 | | 0.6721 |
| PNI | 0.9860 | 0.9553 | 1.0177 | | 0.3837 |
| Hemoglobin (g/L) | 0.9908 | 0.9784 | 1.0034 | | 0.1502 |
| D-2 polymer (mg/L) | 1.0266 | 0.9625 | 1.0949 | | 0.4250 |
| Troponin T ( ng/L) | 1.0001 | 0.9994 | 1.0007 | | 0.9103 |
| Serum NT-proBNP (pg/mL) | 1.0001 | 1.0000 | 1.0001 | | 0.0605 |
| Left ventricular ejection fraction (%) | 0.9659 | 0.9437 | 0.9886 | | 0.0034 |
| Surgical types | | | | | |
| Coronary artery bypass graft surgery | 1.1247 | 0.7030 | 1.7994 | | 0.6239 |
| valve surgery | 1.1228 | 0.6964 | 1.8103 | | 0.6345 |
| Ascending aorta or aortic arch replacement | 1.5316 | 0.8434 | 2.7815 | | 0.1613 |
| Coronary artery bypass graft surgery + valve surgery | 1.9702 | 0.9461 | 4.1028 | | 0.0700 |
| Corrective surgery for congenital heart disease | 0.5658 | 0.1674 | 1.9129 | | 0.3595 |
| Intraoperative information | | | | | |
| Intraoperative bleeding volume (ml) | 1.0003 | 0.9996 | 1.0010 | | 0.4168 |
| Surgical duration (h) | 1.3225 | 1.1530 | 1.5168 | | 0.0001 |
| Aortic cross-clamp time (h) | 1.7559 | 1.6914 | 1.8204 | | 0.0700 |
| Cardiopulmonary bypass time (h) | 2.3520 | 2.2803 | 2.4238 | | 0.1670 |
| Total intraoperative fluid input (ml) | 1.0002 | 1.0001 | 1.0003 | | 0.0048 |
| Total intraoperative fluid output (ml) | 1.0002 | 1.0001 | 1.0003 | | 0.0044 |
| VIS at the beginning of the surgery (score) | 1.0111 | 0.9301 | 1.0991 | | 0.7958 |
| VIS at the end of the surgery (score) | 1.0549 | 0.9740 | 1.1425 | | 0.1891 |
| Highest VIS during the surgery (score) | 1.0644 | 0.9904 | 1.1440 | | 0.0894 |
| Postoperative information | | | | | |
| Postoperative use of recombinant human brain natriuretic peptide | 1.2872 | 0.7715 | 2.1475 | | 0.3337 |
| Postoperative use of nitroglycerin | 0.6269 | 0.3883 | 1.0120 | | 0.0560 |
| Mechanical ventilation duration before weaning (h) | 1.0136 | 1.0086 | 1.0187 | | ＜0.0001 |
| Serum NT-proBNP on weaning day (pg/mL) | 1.0001 | 1.0001 | 1.0002 | | ＜0.0001 |
| Highest lactate level before weaning (mmol/L) | 1.1087 | 1.0171 | 1.2086 | | 0.0190 |
| mSOFA on weaning day (score) | 1.7451 | 1.4768 | 2.0620 | | ＜0.0001 |
| WBC count on weaning day (10^9^/L) | 1.0262 | 0.9792 | 1.0754 | | 0.2799 |
| Hemoglobin on weaning day (g/L) | 0.9797 | 0.9650 | 0.9945 | | 0.0075 |
| D-dimer on weaning day (mg/L) | 1.0652 | 1.0177 | 1.1149 | | 0.0066 |
| Troponin T on weaning day (ng/L) | 1.0002 | 1.0000 | 1.0004 | | 0.0447 |
| Procalcitonin on weaning day (ng/mL) | 1.0954 | 1.0404 | 1.1534 | | 0.0005 |
| Fastest heart rate before weaning (beats/min) | 1.0089 | 0.9915 | 1.0265 | | 0.3194 |
| Highest mean arterial pressure before weaning (mmHg) | 0.9983 | 0.9871 | 1.0097 | | 0.7735 |
| Highest central venous pressure before weaning (cmH^2^O) | 1.0127 | 0.9738 | 1.0531 | | 0.5278 |
| VVR on admission to ICU (score) | 1.0452 | 1.0167 | 1.0744 | | 0.0017 |
| VVR before weaning (score) | 1.2096 | 1.1542 | 1.2677 | | ＜0.0001 |
| Highest VVR before weaning (score) | 1.0633 | 1.0394 | 1.0877 | | ＜0.0001 |

BMI, Body mass index; WBC, White blood cell; PNI, Prognostic Nutritional Index; NT-proBNP, N-terminal pro-B-type natriuretic peptide; mSOFA, modified Sequential Organ Failure Assessment; VIS, vasoactive-inotropic score; VVR, vasoactive-ventilation-renal score.
