# Supplementary figures and images for: Prediction of ventilator weaning failure in postoperative cardiac surgery patients using vasoactive-ventilation-renal score and nomogram analysis
Source: Front Cardiovasc Med. 2024 Mar 14;11:1364211. doi: 10.3389/fcvm.2024.1364211 (PMC10977076; doi:10.3389/fcvm.2024.1364211)

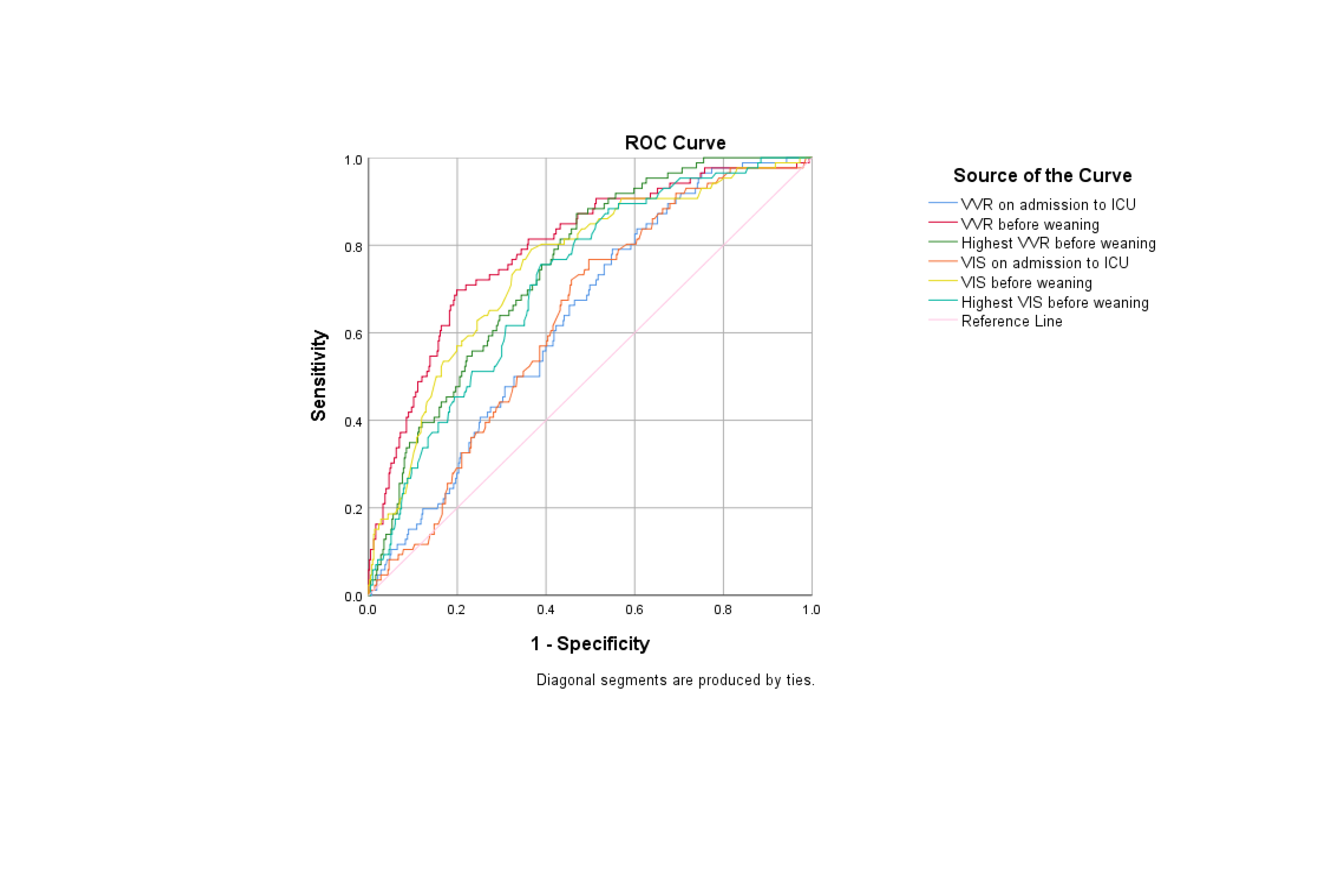

Supplement: Supplementary file 1 [file Datasheet1.zip › Data Sheet 1_v1/Figure S1.tif]

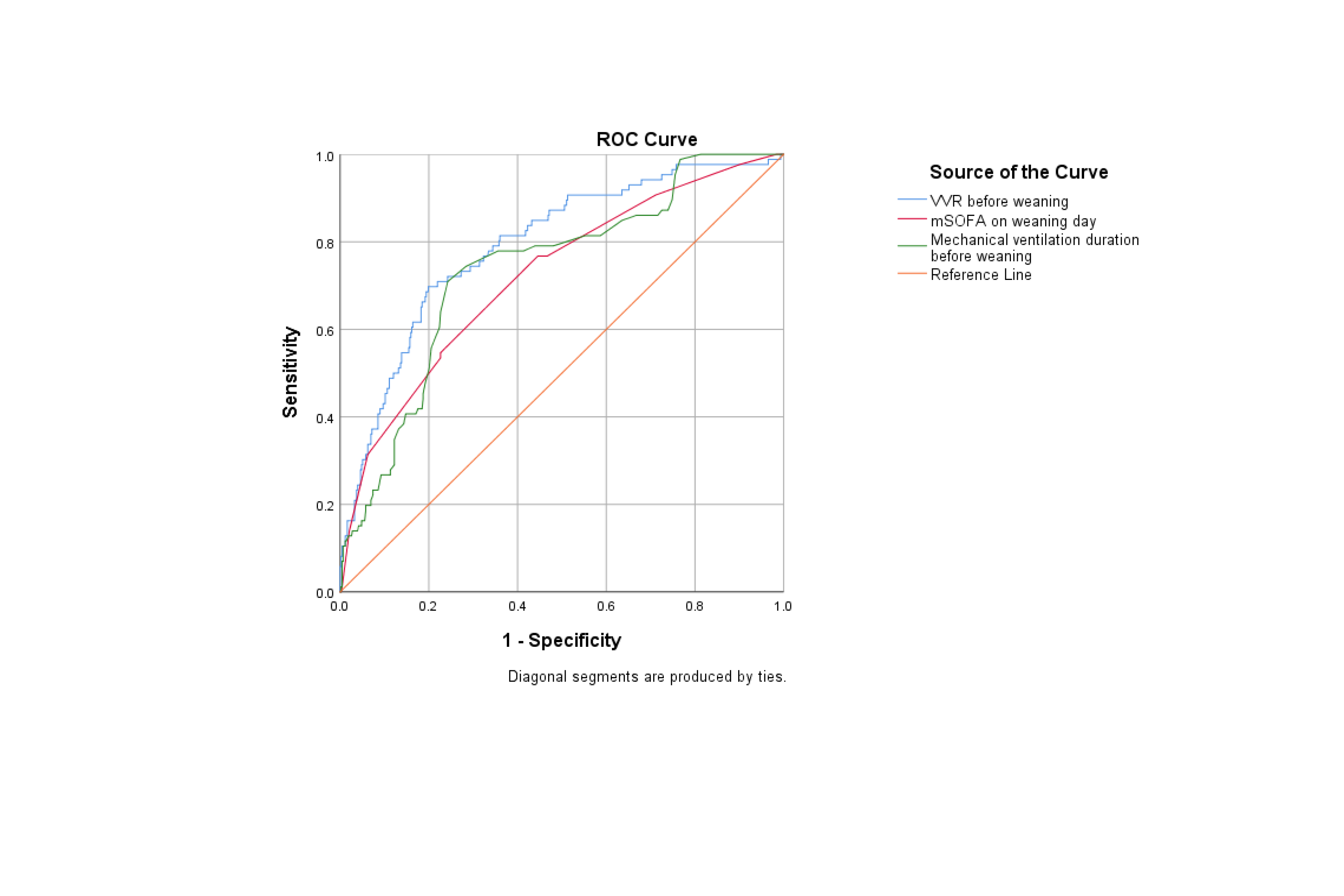

Supplement: Supplementary file 1 [file Datasheet1.zip › Data Sheet 1_v1/Figure S2.tif]

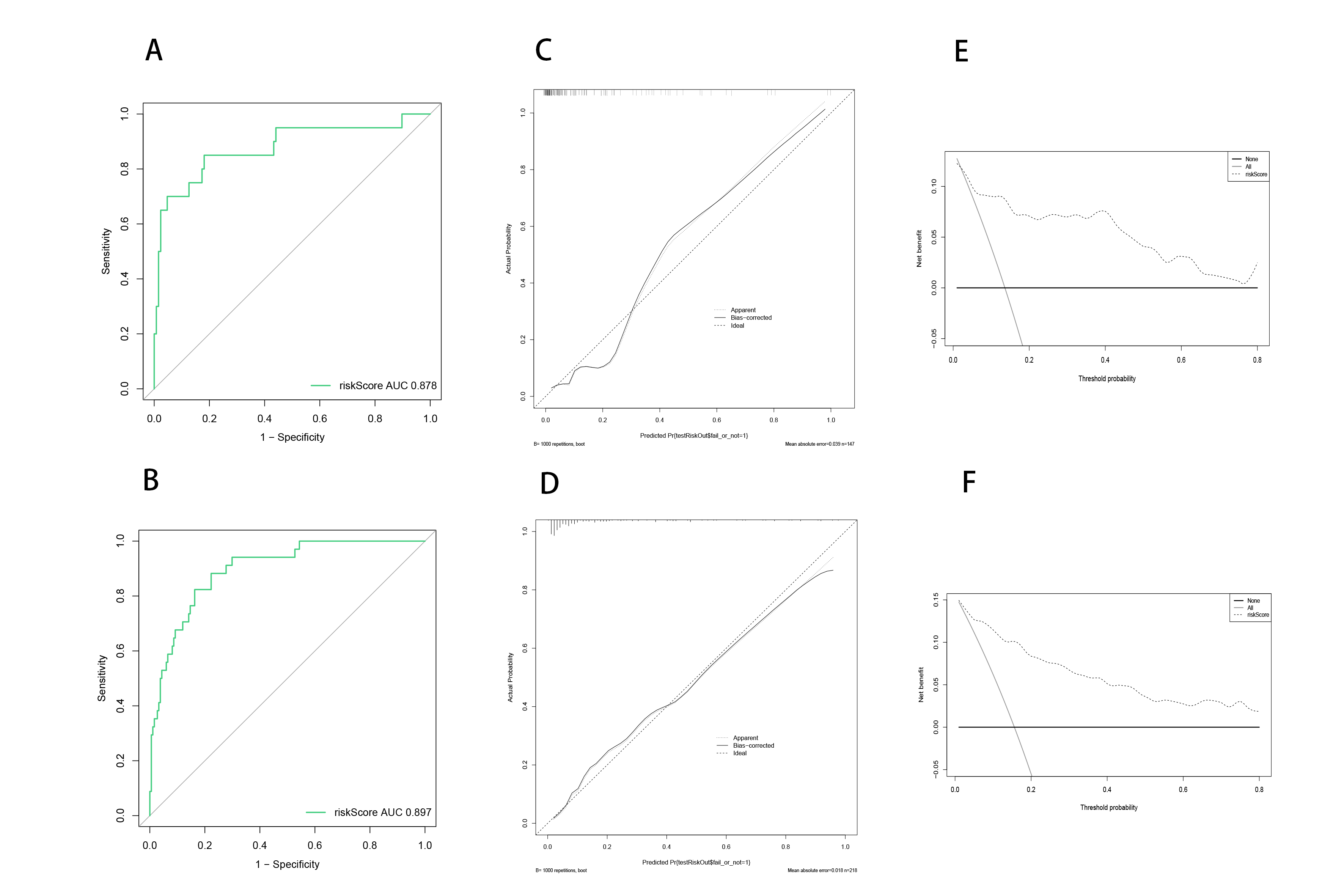

Supplement: Supplementary file 1 [file Datasheet1.zip › Data Sheet 1_v1/Figure S3.tif]
